# Presence, Mode of Action, and Application of Pathway Specific Transcription Factors in *Aspergillus* Biosynthetic Gene Clusters

**DOI:** 10.3390/ijms22168709

**Published:** 2021-08-13

**Authors:** Wenjie Wang, Yuchao Yu, Nancy P. Keller, Pinmei Wang

**Affiliations:** 1 Ocean College, Zhejiang University, Zhoushan 316021, China; 21534024@zju.edu.cn (W.W.); yuyuchao@zju.edu.cn (Y.Y.); 2 Department of Medical Microbiology and Immunology, University of Wisconsin-Madison, Madison, WI 53706, USA; 3 Department of Bacteriology, University of Wisconsin-Madison, Madison, WI 53706, USA

**Keywords:** pathway specific transcription factor, transcription factor, *Aspergillus*, regulation, evolution

## Abstract

Fungal secondary metabolites are renowned toxins as well as valuable sources of antibiotics, cholesterol-lowering drugs, and immunosuppressants; hence, great efforts were levied to understand how these compounds are genetically regulated. The genes encoding for the enzymes required for synthesizing secondary metabolites are arranged in biosynthetic gene clusters (BGCs). Often, BGCs contain a pathway specific transcription factor (PSTF), a valuable tool in shutting down or turning up production of the BGC product. In this review, we present an in-depth view of PSTFs by examining over 40 characterized BGCs in the well-studied fungal species *Aspergillus nidulans* and *Aspergillus fumigatus*. Herein, we find BGC size is a predictor for presence of PSTFs, consider the number and the relative location of PSTF in regard to the cluster(s) regulated, discuss the function and the evolution of PSTFs, and present application strategies for pathway specific activation of cryptic BGCs.

## 1. Introduction

Secondary metabolites (SMs) in fungi, also known as natural products, are present and synthesized in ecologically diverse species [1]. In fungi, SMs can provide self-protection and act as mediators for communication with other organisms or as virulence factors during pathogenic interactions with plants and animals [2,3,4,5,6]. Many SMs were proven to be associated with potentially useful biologic activities [7,8]. Further, some SMs are able to promote health and longevity [9]. 

Global aging and the variety of both infectious and non-infectious illnesses created an urgent need for discovery of new drug leads. Over the last 90 years, medicinal properties of fungal SMs, such as penicillin, cyclosporin, and statins, were utilized for various health therapies [10,11,12]. However, thus far, only a small subset of fungal SMs is characterized, despite the realization that fungi contain 100,000 s to millions of SM biosynthetic gene clusters (BGCs) [1,4]. The growing interest in fungal SMs is to discover new drugs and to take control of their production. Therefore, novel solutions of activating/regulating “cryptic” BGCs must be developed [13,14]. Hence, an important goal in mining fungal SMs is to characterize the molecular mechanisms of their production [15].

*Aspergillus* spp., with over 330 species, represents a major fungal genus with potent SM arsenal [4]. *Aspergillus* SMs are usually derived from polyketide synthases (PKSs), nonribosomal peptide synthetases (NRPSs), PKS/NRPS hybrids, terpene cyclases (TCs), dimethylallyltryptophan synthases (DMATSs), and isocyanide synthases (ICSs) [8,16]. Typically, a BGC is minimally composed of a synthase that uses primary metabolites to form carbon backbone that is further modified by tailoring enzymes, such as methyltransferases, P450 monooxygenases, hydroxylases, and epimerases [4]. Some BGCs contain a gene that encodes a protein involved in resistance/protection mechanisms to mitigate the toxic property of the SM [2]. Moreover, some BGCs contain a pathway specific transcription factor (PSTF) that specifically regulates expression of the genes within the BGC [17]. 

Transcription factors (TFs) are sequence-specific DNA-binding proteins required to modulate gene expression [18,19]. Recent advances shed light on hierarchical levels of SM transcriptional elements by highlighting the role of global regulators (e.g., the Velvet complex [20,21]), stress response regulators (e.g., PacC mediating fungal response to pH [22]), epigenetic regulators (e.g., the COMPASS complex in *A. nidulans* [23,24]), as well as a variety of TFs including pathway specific transcription factors (PSTFs). Whereas all of these elements are utilized for genome mining of fungal BGCs, the use of PSTFs presents the most mechanistically clear approach. However, in many cases, PSTF overexpression does not result in successful BGC activation [25]. Therefore, understanding how PSTFs regulate their biosynthetic genes is critical to the development of new strategies to discover “cryptic” SMs as potential drug molecules.

For a better understanding of PSTFs, it is essential to know the entire repertoire of PSTFs in a species. Although no one species has the entirety of its BGCs characterized, two *Aspergillus* species, *A. nidulans* and *A. fumigatus*, are closing in a full analysis with 28 defined BGCs in the former [26,27] and 18 defined BGCs in the latter [4,28,29,30]. In this review, we examine these two model fungi and identify which BGCs contain PSTFs, characterize known regulatory mechanisms of each PSTF, and present recommendations on how to better utilize PSTFs for the discovery of novel SMs.

## 2. Occurrence and Types of Pathway Specific Transcription Factors

Currently, 31 core synthase genes contained in 28 BGCs in *A. nidulans* (Table 1) and 23 core synthase genes contained in 18 BGCs in *A. fumigatus* (Table 2) are identified along with their downstream SMs. Some BGCs contain more than one synthase gene, e.g., *inpA* and *inpB* are two NRPS genes in *A. nidulans* fellutamide B BGC [31], and *fgaPT1* (DMATS), *pes1* (NRPS), and *pesL* (NRPS) are essential for the production of fumigaclavine C in *A. fumigatus* [32,33]. Among these known BGCs, 12 BGCs (42.3%) contain 16 PSTFs in *A. nidulans,* and 10 BGCs (55.6%) contain 12 PSTFs in *A. fumigatus*. 

The number of genes that are involved in the production of SMs can vary greatly. Some SMs need only a single gene, e.g., PKS8 is a stand-alone gene in *Fusarium graminearum* responsible for production of gibepyrones and prolipyrone B [34]. Most SMs need the coordinated involvement of few to many gene products for the assembly of the BGC product [35]. For example, the alternariol cluster of *A. nidulans* comprises two genes, a non-reducing PKS gene *pkgA* and a β-lactamase-type thioesterase gene *pkgB* [36], while the sterigmatocystin cluster of *A. nidulans* consists of 25 genes [37]. The size of the BGC is related to the occurrence of PSTF. In *A. nidulans* and *A. fumigatus*, most PSTFs are located in BGCs with more than five genes. Among the BGCs with more than five genes, more than two third of them contain PSTFs—12 out of 15 (80.0%) BGCs in *A. nidulans* and 9 out of 14 (64.3%) BGCs in *A. fumigatus* (Table 3). Possibly, this association of PSTF with larger BGC underlies a need for co-regulation of multiple genes that cannot be achieved by broader *cis*-regulatory networks.

TFs can be classed into families based on their DNA-binding domains (DBDs), including 37 PFAM families, and the largest class of fungal-specific domains is the zinc-cluster superfamily [19]. In *A. nidulans*, PSTFs belong to three families of TF DBDs—Zn(II)_2_Cys_6_ type, C2H2 type, and Myb-like DNA-binding domain type—while several PSTFs do not have a conserved domain, and two contain fungal specific transcription factor domains (Table 1). In *A. fumigatus*, PSTFs belong to two families of TF DBD—Zn(II)_2_Cys_6_ type and bZIP type—while TpcD is a homolog of AflS without a conserved domain (Table 2). AflS is a PSTF without a conserved domain but has its critical role in the aflatoxin/sterigmatocystin (AF/ST) biosynthetic pathway as a co-activator of AflR [38]. However, other PSTFs without a conserved domain have varying functions, which are discussed later. Four types of PSTFs are involved in the BGCs with six types of synthase genes (NRPS, PKS, PKS/NRPS hybrid, DMATS, TC, and ICS) (Table 1 and Table 2). We found no significant relationship between BGC types and PSTF types.

## 3. Role and Evolution of Pathway Specific Transcription Factor (PSTF)

Of those BGCs containing PSTFs, most contain one PSTF, but there are variations, including BGCs with two PSTFs or a single PSTF that regulates more than one BGC (Table 4); examples are discussed below. Additionally, most PSTFs are positive regulators with some exceptions: one of the *A. fumigatus* hexadehydroastechrome BGC PSTFs, HasF, has no apparent function [88], and the second PSTF of the *A. nidulans* felinone A BGC, DbaG, is a negative regulator but under the control of the positive PSTF DbaA [39,40].

### 3.1. One PSTF per BGC

#### 3.1.1. PSTF with Single Conserved Function

**Figure 1:**
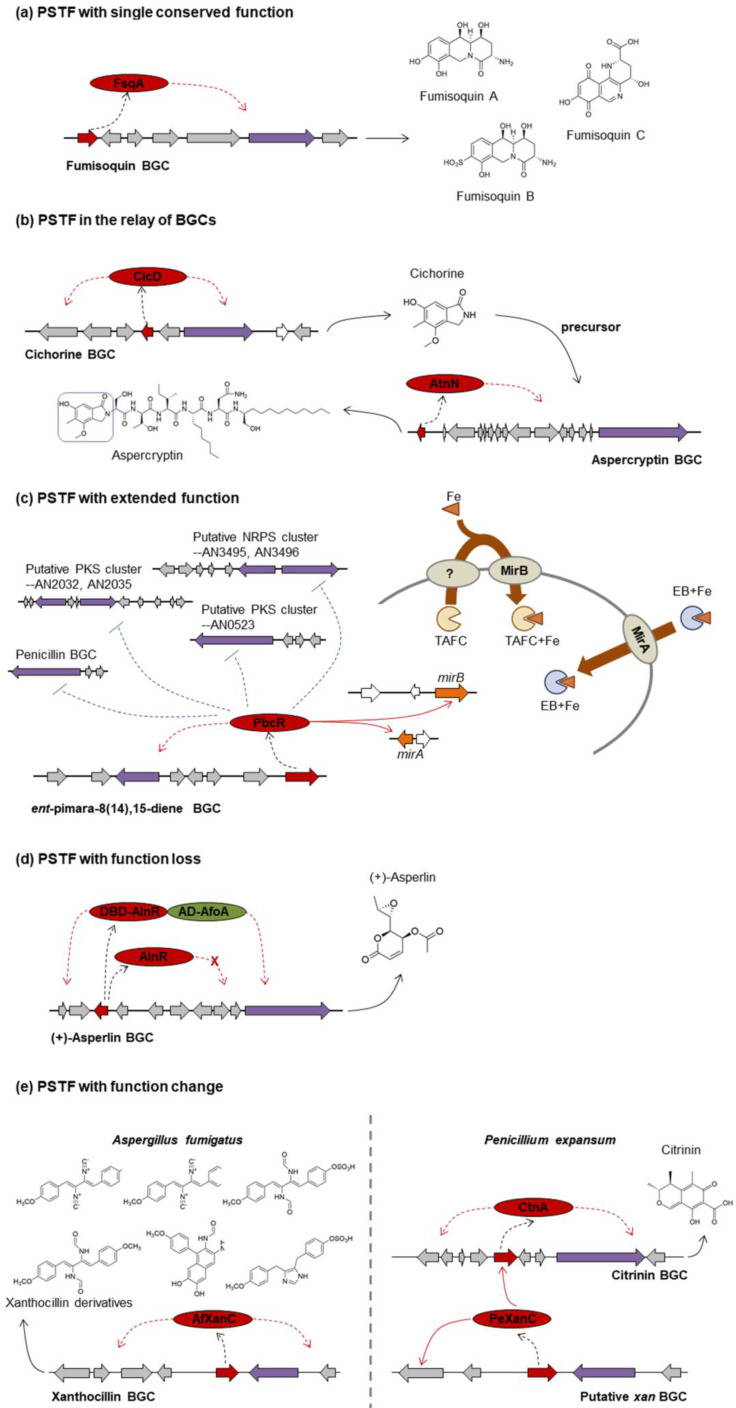
Examples of “one PSTF per BGC”. (**a**) PSTF with single conserved function. (**b**) PSTF in the relay of BGCs. (**c**) PSTF with extended function. (**d**) PSTF with function loss. AD-AfoA (green) means the activation domain (AD) from AfoA. DBD-AlnR (red) means the DNA-binding domain (DBD) from AlnR. (**e**) PSTF with function change. Purple indicates backbone genes; red indicates positive PSTFs; grey indicates tailoring genes involved in the SM biosynthetic pathway; white indicates genes not involved in the SM biosynthetic pathway; orange indicates *mirA* and *mirB* genes under the regulation of PSTF PbcR and responsible for transporting iron. The dotted black arrow indicates the process of protein translation by the PSTF gene; the solid black arrow indicates the process of SM(s) production by the BGC; the dotted red arrow indicates the positive regulation by the PSTF for all the other biosynthetic genes in the cluster; the solid red arrow indicates positive regulation by the PSTF for the specific gene; the dotted blue arrow with a stop end indicates the negative regulation by the PSTF for the whole cluster genes. (**e**) is based on Figure 6 in reference [95]. PSTF = pathway specific transcription factor, BGC = biosynthetic gene cluster.

The basic function of the single PSTF in a single BGC is as a specific positive regulator. Overexpression of this type of PSTF is useful in inducing cryptic/low production metabolites or identifying the boundaries of the BGC. For example, inducing expression of the PSTF gene *apdR* activates the cryptic PKS/NRPS hybrid gene cluster to produce aspyridones A and B [49], and overexpression of the positive PSTF gene *fsqA* regulates expression of six adjacent genes, *fsqB* to *fsqG*, and defines the boundaries of the *fsq* cluster which is responsible for fumisoquin biosynthesis [80] (Figure 1a). Overexpression of *nscR* leads to isolation of a new metabolite neosartoricin [91]. The PSTF HmgR is a transcriptional activator for the genes of the tyrosine degradation cluster, which is a different pathway from DHN-melanin and produces an alternative melanin, pyomelanin [93].

#### 3.1.2. PSTF in the Relay of BGCs: *cicD*, *atnN*

The cichorine BGC contains seven genes identified by a set of targeted deletions [51]. Deletion of the PSTF gene *cicD* eliminates the production of cichorine, which indicates that CicD is a positive PSTF. Interestingly, cichorine is the precursor of aspercryptin [41]. In other words, aspercryptin is produced by two distinct clusters that are physically separated in the genome, the *cic* cluster (in chromosome I) and the *atn* cluster (in chromosome II) (Figure 1b). The core biosynthetic gene for the cichorine pathway is a PKS, and the core biosynthetic gene for the aspercryptin cluster is an NRPS (Table 1). Similar to *cicD* in the *cic* cluster, the *atn* cluster also has a positive PSTF gene, *atnN*, and overexpression of *atnN* leads to increased aspercryptin A1 production [42]. This raises the exciting possibility that *A. nidulans* uses PSTFs to regulate different SM gene clusters to expand their repertoire of natural products and tailor the SM arsenal to achieve maximum competitive advantage.

#### 3.1.3. PSTF with Extended Function: *pbcR*

Overexpression of the PSTF gene *pbcR* upregulates the transcription of seven genes in the identified cluster and leads to the production of a diterpene compound, *ent*-pimara-8(14),15-diene [54]. Additionally, the expression levels of siderophore transporter genes *mirA* and *mirB* are upregulated. Further, overexpression of *pbcR* downregulates four other clusters (penicillin cluster, two putative PKS clusters, and one putative NRPS cluster) (Figure 1c), which might be a way for *A. nidulans* to reserve sufficient primary metabolites for cell growth or for the specific production of *ent*-pimara-8(14),15-diene [54]. However, the mechanism for the downregulation of these clusters in the *pbcR* overexpression strain is still not clear, and a possible function of *pbcR* in iron homeostasis is waiting to be explored. These studies on *pbcR* indicate that one extending function of PSTF could be in linking with higher-level regulation system(s) or other SMs.

#### 3.1.4. PSTF with Function Loss: *alnR*

Replacement of the promoter of PSTF gene *alnR* with the inducible *alcA* promoter did not result in any detectable product. However, when Grau et al. [45] fused the DNA-binding domain (DBD) of AlnR with the activation domain (AD) from AfoA, the hybrid PSTF activated transcription of the target BGC genes to obtain the antibiotic (+)-asperlin (Figure 1d). This gives an example of function loss of PSTF due to the incomplete/non-functional domains, which might be the cause of the failure of other studies that were unsuccessful in overexpressing PSTFs to activate cryptic BGCs.

#### 3.1.5. PSTF with Function Change: *xanC*

In *A. fumigatus*, overexpression of *xanC* upregulates the expression of all xanthocillin biosynthesis genes and increases abundance of all downstream xanthocillin derivatives [16], which indicates XanC (AfXanC) is a positive PSTF for the *xan* cluster. A highly conserved *xan* cluster with a *xanC* homolog (*PexanC*) was identified in *Penicillium expansum*. Surprisingly, instead of regulating the *Pexan* BGC (with the exception of one gene, *PexanG*), PeXanC activates the citrinin PSTF gene *ctnA* and increases the production of citrinin [95] (Figure 1e). This divergence is partially explained by the finding that AfXanC and PeXanC recognize different DNA binding sites [95]. This gives an example of evolutionary variance and functional change of PSTF homologs. 

### 3.2. Two PSTFs per BGC

In *A. nidulans* and *A. fumigatus*, one quarter of PSTF-containing BGCs have two PSTFs in a single BGC. Interestingly, instead of playing the similar function of regulating genes, one TF usually plays the predominant positive role, while the other TF plays a variant function, e.g., co-activator, negative regulator, or no function at all. Moreover, *mdpE* and *mdpA* present an example of a more complex regulatory scheme, as discussed below. 

#### 3.2.1. AflR-AflS Type

- *aflR*-*aflS*

**Figure 2:**
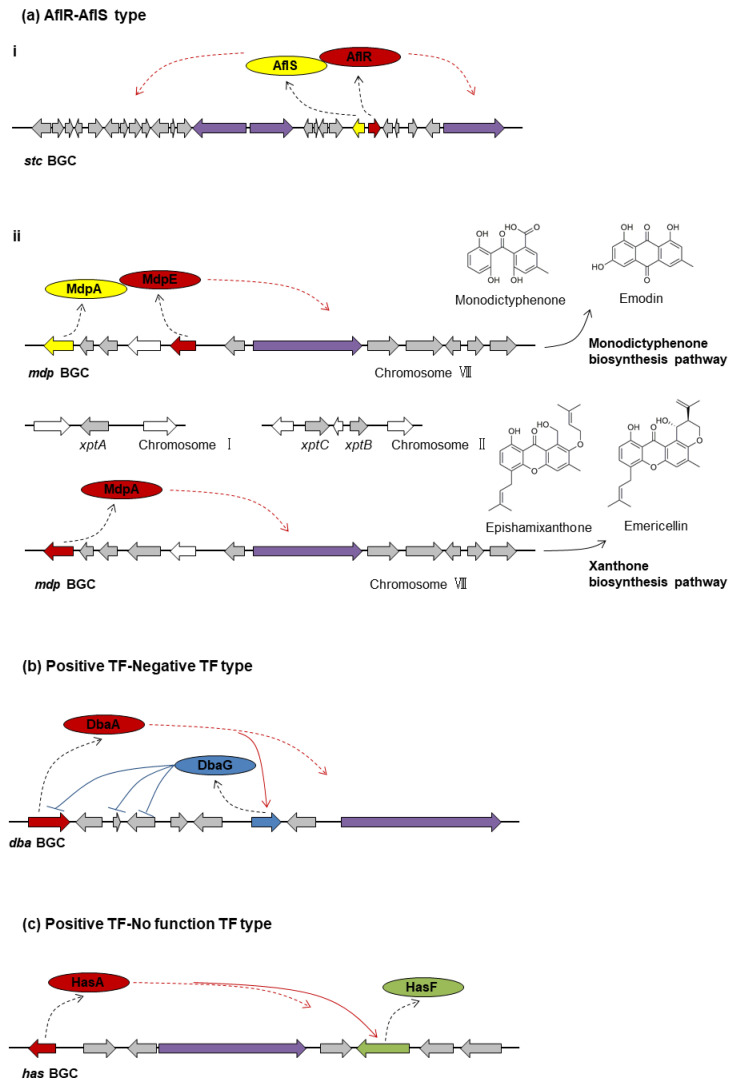
Examples of “two PSTFs per BGC”. (**a**) AflR-AflS type. (**i**) *aflR*-*aflS* in the sterigmatocystin (ST) BGC; (**ii**) *mdpE*-*mdpA* in monodictyphenone or xanthone biosynthesis. (**b**) Positive TF-Negative TF type. (**c**) Positive TF-No function TF type. Purple indicates backbone genes; red indicates positive PSTFs; yellow indicates the second PSTF gene as co-activator; blue indicates negative PSTF; green indicates PSTF with no function; grey indicates tailoring genes involved in the SM biosynthetic pathways; white indicates genes not involved in the SM biosynthetic pathway. The dotted black arrow indicates the process of protein translation by the PSTF gene; the solid black arrow indicates the process of SM(s) production by the BGC; the dotted red arrow indicates the positive regulation by the PSTF for all the other biosynthetic genes in the cluster; the solid red arrow indicates positive regulation by the PSTF for the specific gene; the solid blue arrow with a stop end indicates the negative regulation by the PSTF for the specific gene.

The aflatoxin/sterigmatocystin (AF/ST) gene cluster represents one of the best characterized mycotoxin gene clusters with two PSTF genes, *aflR* and *aflS* (previously named as *aflJ*). AflR is a Zn(II)_2_Cys_6_ TF, while AflS is a TF without a conserved domain but shows some low similarity to a methyltransferase. AflR activates the transcription of most structural genes in the ST gene cluster in *A. nidulans*, and deletion of *aflR* abolishes the ST synthesis [96]. AflS plays a role in the regulation of the ST biosynthesis through interacting with AflR and is often termed as a co-activator. The Δ*aflS* mutant produces reduced but detectable levels of ST [96] (Figure 2a(i)). *A. flavus aflR* is able to complement the *A. nidulans* Δ*aflR* strain and restore the ST production [97]. However, *A. sojae* is unable to produce AF/ST due to a functional mutation of *aflR*. The mutation in *aflR* results in a truncated protein and the failure of AflR to interact with AflS [98].

- *mdpE*-*mdpA*

*mdpE* and *mdpA* encode PSTFs regulating expression of *mdp* cluster genes responsible for the production of monodictyphenone and prenyl xanthones in *A. nidulans* [61,62]. MdpE is a distinct Zn(II)_2_Cys_6_ TF homologous to AflR, while MdpA is a TF homologous to AflS. Inducing expression of *mdpE,* but not *mdpA,* activated expression of *mdp* cluster genes, resulting in the production of monodictyphenone and emodin analogs [23,62]. Similar to the AflR-AflS regulation model, MdpE is the positive acting DNA binding partner, while MdpA is the co-activator in the monodictyphenone biosynthesis. 

Some *mdp* cluster genes also participate in the prenyl xanthone biosynthesis with emodin and monodictyphenone precursors for producing prenyl xanthones [61]. Unexpectedly, *mdpD* (encoding a monooxygenase) and *mdpE* (encoding a PSTF) are not consistently involved in monodictyphenone biosynthesis and xanthone biosynthesis. *mdpD* is unnecessary for monodictyphenone generation but is required for xanthone synthesis, whereas *mdpE* is necessary for monodictyphenone generation but is not required for xanthone synthesis [61]. In contrast to *mdpE*, the PSTF gene *mdpA* is required for the synthesis of both monodictyphenone and xanthone [61] (Figure 2a(ii)).

- *tpcE*-*tpcD*

In *A. fumigatus*, there is an unusual example of two physically discrete BGCs (endocrocin cluster and trypacidin cluster) generating the same SM—endocrocin [72]. The *tpc* cluster makes use of different gene combinations to produce either endocrocin or trypacidin. Trypacidin is eliminated when only *tpc* cluster genes are deleted, whereas the elimination of endocrocin production needs the deletion of both *tpc* cluster and *enc* cluster genes. A homolog of AflR, TpcE, is required for regulating *tpc* genes to produce trypacidin and endocrocin with a co-activator, TpcD, which is an AflS homolog. No PSTF exists in the *enc* cluster, which contains four genes in total [72].

#### 3.2.2. Positive TF-Negative TF Type: *dbaA*-*dbaG*

Overexpression of the PSTF gene *dbaA* coordinately upregulates nine consecutive genes, which defines the boundaries of the *dba* cluster in *A. nidulans* to produce 2,4-dihydroxy-3-methyl-6-(2-oxopropyl)benzaldehyde (DHMBA) [40]. However, overexpression of another PSTF gene in the *dba* cluster, *dbaG*, slightly increases the expression of one gene (*dbaF*) but decreases the expression levels of three other genes (*dbaA*, *dbaC,* and *dbaD*) [40]. Different from positive TF DbaA, DbaG is a negative PSTF (Figure 2b). Interestingly, *dbaG* is under control of the positive acting PSTF DbaA, suggesting a complex transcriptional control of the entire *dba* gene cluster.

#### 3.2.3. Positive TF-No Function TF Type: *hasA-hasF*

The Zn(II)_2_Cys_6_ TF gene *hasA* was down-regulated in an *A. fumigatus* Δ*laeA* array, leading to the hypothesis that it could be a PSTF for surrounding genes [99]. Indeed, overexpression of *hasA* results in the expression of seven adjacent genes (including the second TF *hasF*) responsible for the production of hexadehydroastechrome (HAS) [88]. However, loss of the second transcription factor HasF (OE::*hasA*Δ*hasF*) does not result in any detectable metabolomic change compared to the OE::*hasA* strain, indicating that HasF does not affect the *has* pathway [88] (Figure 2c).

### 3.3. One PSTF for Two BGCs: fapR

**Figure 3:**
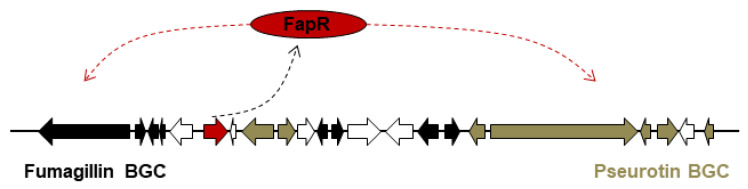
Model of FapR in a supercluster containing fumagillin and pseurotin biosynthetic genes. Black indicates genes involved in the fumagillin biosynthesis; brown indicates genes involved in the pseurotin biosynthesis; red indicates the positive PSTF gene *fapR*; white indicates genes not involved in either biosynthetic pathway. The dotted black arrow indicates the process of protein translation by the PSTF gene *fapR*; the dotted red arrow indicates the positive regulation by the PSTF for all the biosynthetic genes in the two clusters.

In the telomeric region of chromosome VIII in *A. fumigatus*, there is a supercluster containing two intertwined BGCs that produce fumagillin [75,76] and pseurotin A [92], respectively (Figure 3). They do not share any structural genes/enzymes for their syntheses, however, fumagillin and pseurotin biosynthetic genes are physically interspersed [77]. The Zn(II)_2_Cys_6_ TF FapR (also called FumR [76]) was confirmed to regulate both pseurotin and fumagillin cluster genes [77].

### 3.4. Additional Complexities in PSTF Regulation

#### 3.4.1. PSTF in Cross Talk: *scpR-afoA*

**Figure 4:**
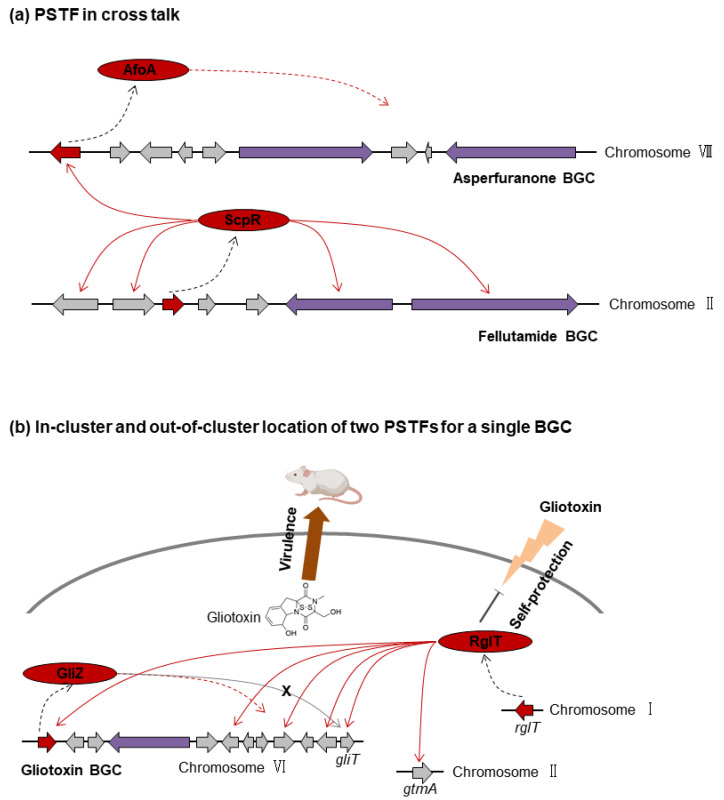
Examples of additional complexities in PSTF regulation. (**a**) PSTF in BGC cross talk. (**b**) In-cluster and out-of-cluster locations of two PSTFs regulating a single BGC. Purple indicates backbone genes; red indicates positive PSTF genes; grey indicates tailoring genes involved in the SM biosynthetic pathways. The dotted black arrow indicates the process of protein translation by the PSTF gene; the dotted red arrow indicates the positive regulation by the PSTF for all the other biosynthetic genes in the cluster; the solid red arrow indicates positive regulation by the PSTF for the specific gene; the solid grey arrow with an “X” indicates that *gliT* is not under the regulation of the PSTF GliZ. (**a**) is based on this figure in reference [44].

In chromosome II in *A. nidulans*, the silent *inp* cluster contains two NRPS genes, *inpA* and *inpB*, flanked by the PSTF gene *scpR*. The induced expression of *scpR* leads to the transcriptional activation of *inpA* and *inpB*, the fatty-acyl-AMP ligase gene *inpC,* and the transporter gene *inpD* but not the activation of the proteasome gene *inpE* or the release gene *inpF* [44]. No NRPS product was detected from *scpR* overexpression because *inpE* and *inpF* were confirmed in further research to be essential for the final product of *inp* cluster, a proteasome inhibitor fellutamide B [31]. The proteasome InpE is required for resistance to the intracellularly produced fellutamide B. Interestingly, instead of activating all six *inp* genes to produce fellutamide B, ScpR activates the silent *afo* cluster in chromosome VIII through activating the *afo* cluster PSTF gene *afoA* to produce the polyketide asperfuranone [44] (Figure 4a). The cross talk between ScpR and AfoA is similar to the previously mentioned regulation of the *cit* cluster by PeXanC in *P. expansum*, with both presenting instances of unexpected divergence of PSTF target pathways.

#### 3.4.2. In-Cluster and Out-of-Cluster Locations of Two PSTFs for a Single BGC: *gliZ* and *rglT*

Gliotoxin is known as a mycotoxin and a virulence factor in *A. fumigatus* [100]. GliZ, a positive PSTF, is indispensable for the gliotoxin biosynthesis. The overexpression of *gliZ* increases gliotoxin production, and *gliZ* deletion greatly reduces gliotoxin production and eliminates the expression of some other *gli* cluster genes (e.g., *gliI*, *gliA*, *gliG*) but not *gliT* (encoding a thioredoxin reductase that protects the fungus from gliotoxin) [101,102]. Interestingly, *gliT* is under the regulation of RglT, another PSTF regulating the *gli* BGC but located outside of the cluster (Figure 4b) [86]. RglT regulates the expression of *gliF*, *gliM*, *gliT,* and *gliA* as well as *gtmA* (not in *gli* cluster) in the presence of allyl alcohol and of *gliZ*, *gliT,* and *gliF* under the gliotoxin-inducing conditions through directly binding to these gene promoter regions [86]. The ∆*rglT* strain cannot produce gliotoxin, indicating that RglT is essential for the gliotoxin biosynthesis. RglT mediates *A. fumigatus* self-protection against exogenous gliotoxin, probably through the expression of *gliT* and the bisthiomethyltransferase-encoding gene *gtmA*. This gives an example of a single BGC containing one in-cluster PSTF and another out-of-cluster PSTF, both involved in virulence but only one activating self-protection mechanisms.

## 4. Pathway Specific Approaches to Explore Biosynthetic Gene Clusters

A number of useful approaches were developed to activate cryptic BGCs, and the most rapid, easy-handling, and versatile method is to manipulate the PSTF. It often allows for precise, controllable activation of a specific BGC and expectations to unambiguously identify metabolic products. 

### 4.1. Overexpression/Deletion of PSTF

Most PSTFs are positive-regulators, while few of them are negative-regulators, as discussed previously. Thus, inducing the expression of pathway specific activator genes and deleting pathway specific repressor genes are both promising approaches for activating cryptic BGCs [103]. To overexpress positive acting PSTFs, replacing the native promoter with a constitutive promoter or an inducible promoter is commonly used. For example, overexpression of the PSTF gene *fsqA* using the constitutive promoter *gpdAp* from *A. nidulans* upregulated the expression of the *fsq* BGC and produced several new isoquinoline alkaloids known as fumisoquins [80]. Activation of the PSTF gene *apdR* with the inducible alcohol dehydrogenase promoter *alcAp* from *A. nidulans* resulted in expression of the *apd* BGC (*apdA*, *apdB*, *apdC*, *apdD*, *apdE,* and *apdG*) under inducing conditions to produce aspyridones A and B [49].

### 4.2. Synthetic PSTF

The approach of replacing the promoter of PSTF can work spectacularly well, but not always. This might be due to domain function loss of a PSTF, lack of an unknown natural inducer, requirement for a different or second TF, requirement of a specific precursor, or requirement of post-translational modification of a PSTF, among other possibilities [104]. In some cases, synthetic PSTFs circumvented these problems. 

#### 4.2.1. Hybrid PSTF

For the silent *A. nidulans* BGC containing a highly reducing PKS gene, *AN11191*, the normal approach of upregulating the cluster PSTF could not activate this gene cluster [36]. Grau et al. [45] built a hybrid PSTF to drive the expression of this cluster successfully. They added an activation domain (AD) from AfoA, the PSTF that drives expression of the asperfuranone gene cluster, to the in-cluster PSTF AlnR. The hybrid PSTF AlnR(5-407)-AfoA(130–666) contains the DNA binding domain (DBD), consisting of the Zn(II)_2_Cys_6_ zinc binuclear cluster and the heptad repeats coiled-coil dimerization region, from AlnR and a stable activation domain, which is not precisely defined but is located within residues 130–666 of AfoA. The hybrid PSTF was then driven by inducible promoter *alcAp*. Under inducing conditions, strong expression of the hybrid PSTF resulted in the activation of *aln* genes and production of (+)-asperlin, which is the final product of the *aln* cluster.

#### 4.2.2. CRISPR-Mediated Activation System (Artificial PSTF)

The versatility and the programmability of Cas9 make the CRISPR/Cas9 genome editing strategy a revolutionary approach in biological research. Cas9 can modulate transcription without editing a genomic sequence by fusing the enzymatically inactive version of Cas9 (dCas9) with a transcriptional activation domain; this is known as a CRISPR-mediated activation system (CRISPRa) [105]. Furthermore, by concurrently expressing multiple gRNAs (guide RNAs, specific for the gene sequence of choice), several genes could be simultaneously activated with multiplexed CRISPRa. In addition to dCas9, dCas12 (previously known as dCpf1) is another construct used in CRISPRa, where strong synthetic VP64-p65-Rta (VPR) activators are incorporated into the technology [106]. Roux et al. [25] constructed and tested both CRISPR/dLbCas12a-VPR-based and CRISPR/dSpCas9a-VPR-based activation systems to induce the *mic* BGC in *A. nidulans*. Ultimately, the CRISPR/dLbCas12a-based activation system was successful in the activation of three *mic* cluster genes to produce the final product, dehydromicroperfuranone.

## 5. Conclusions and Perspectives

By examining characterized BGCs and their PSTFs in *A. nidulans* and *A. fumigatus*, we find that the majority of in-cluster PSTFs are positive regulators of BGCs, and overexpression of these PSTFs leads to increased titers of specific BGC products. PSTFs are usually found in larger BGCs, typically with those containing more than five genes. This raises questions to be examined in future studies: how did the need for PSTFs arise in larger clusters, and why do smaller BGCs not require a PSTF? Whereas most PSTFs appear to operate as solo regulators for their localized BGC, some PSTFs interact with other PSTFs for BGC activation (e.g., AflR-AflS and similar dual regulators) or evolve to function in out-of-cluster biosynthetic pathways (e.g., PeXanC vs. AfXanC; ScpR; RglT). Understanding and prediction of *trans* regulatory functions of PSTFs could open new avenues for BGC activation. New strategies are always needed to activate cryptic BGCs and their SM products. A more thorough understanding of PSTF regulatory mechanisms coupled with synergistic development of technologies help to promote the development of pathway specific activation approaches (e.g., synthetic PSTFs and CRISPR-mediated activation systems) can propel advances in this area. The molecular mechanism of PSTF protein–DNA interactions is still limited, and a better understanding of these interactions should also contribute to a useful PSTF toolkit [107]. In our long term goal in synthetic biology, PSTFs would act as the conductor of the engineered orchestra designed to produce new valuable drugs.

## Figures and Tables

**Table 1:** Known secondary metabolites and their pathway specific transcription factors in *A. nidulans*.

SM	Backbone Gene	Backbone Gene Type	Gene Number	PSTF Number	PSTF	PSTF Type	Reference
2,4-dihydroxy-3-methyl-6-(2-oxopropyl)benzaldehyde (DHMBA)/felinone A	*dbaI/pkeA* (AN7903)	NR-PKS	9	2	*dbaA*, *dbaG*	Zn(II)_2_Cys_6_, no conserved domain ^1^	[39,40]
6-hydroxy-7-methyl-3-nonylisoquinoline-5,8-dione	*pkiA* (AN3386)	NR-PKS	3	0	/	/	[36]
Alternariol/isocoumarins	*pkgA* (AN7071)	NR-PKS	2	0	/	/	[36]
Aspercryptins	*atnA* (AN7884), *pkbA* (AN6448)	NRPS, NR-PKS	14 + 7 ^2^	1 + 1 ^3^	*atnN, cicD*	Zn(II)_2_Cys_6_, Myb-like DNA-binding domain	[41,42]
Asperfuranone	*afoE* (AN1034), *afoG* (AN1036)	NR-PKS, HR-PKS	7	1 + 1 ^3^	*afoA*, *scpR*	Zn(II)_2_Cys_6_, C2H2 type zinc finger	[43,44]
(+)-Asperlin	*alnA* (AN11191)	HR-PKS	10	1	*alnR*	Zn(II)_2_Cys_6_	[45]
Aspernidine A	*pkfA* (AN3230)	NR-PKS	6	0	/	/	[46]
Asperniduglene A1 and A2	*sdgA/pkjA* (AN1784)	HR-PKS	4	0	/	/	[47]
Asperthecin	*aptA* (AN6000)	NR-PKS	3	0	/	/	[48]
Aspyridone A and B	*apdA* (AN8412)	PKS/NRPS hybrid	8	1	*apdR*	Zn(II)_2_Cys_6_	[49]
Austinol/dehydroaustinol	*ausA* (AN8383)	NR-PKS	4 + 10 ^2^	0	/	/	[50]
Cichorine	*pkbA* (AN6448)	NR-PKS	7	1	*cicD*	Myb-like DNA-binding domain	[51]
Echinocandin B	*aniA*	NRPS	12	0	/	/	[52]
Emericellamides	*easA* (AN2545), *easB* (AN2547)	NRPS, HR-PKS	4	0	/	/	[53]
*ent*-pimara-8(14),15-diene	AN1594	TC	7	1	*pbcR*	Zn(II)_2_Cys_6_	[54]
F-9775 A and B/violaceol I and II/orsellinic acid	*orsA* (AN7909)	NR-PKS	3	0	/	/	[55,56]
Fellutamide B	*inpA* (AN3495), *inpB* (AN3496)	NRPS, NRPS	6	1	*scpR*	C2H2 type zinc finger	[31]
Ferricrocin	*sidC* (AN0607)	NRPS	3	0	/	/	[57]
Grey-brown conidiophore pigment	*ivoA* (AN10576)	NRPS	2 + 1 ^2^	0	/	/	[58]
4′-Methoxyviridicatin	*asqK* (AN9226)	NRPS	14	1	*asqA*	Fungal specific transcription factor domain ^4^	[59]
Microperfuranone/dehydromicroperfuranone	*micA* (AN3396)	NRPS-like	3	0	/	/	[60]
Monodictyphenone, emodin, Xanthones, Arugosin A and H, Sanghaspirodins A and B	*mdpG* (AN0150)	NR-PKS	1 + 2 + 10 ^2^	2	*mdpE, mdpA*	Zn(II)_2_Cys_6_, no conserved domain ^1^	[23,61,62,63]
Nidulanin A	*nlsA* (AN1242)	NRPS	1 + 1 ^2^	0	/	/	[64]
Penicillin	*acvA* (AN2621)	NRPS	3	0	/	/	[65]
Sterigmatocystin	*stcA/pksST* (AN7825)	NR-PKS	25	2	*aflR*, *aflS*/*aflJ*	Zn(II)_2_Cys_6_, no conserved domain ^1^	[37,64]
Terrequinone A	*tdiA* (AN8513)	NRPS-like	5	0	/	/	[66]
Viridicatumtoxin ^5^	*vrtA*	NR-PKS	13	2	*vrtR1*, *vrtR2*	Fungal specific transcription factor domain ^4^, Zn(II)_2_Cys_6_	[67,68]
YWA1	*wA* (AN8209)	NR-PKS	2	0	/	/	[69]

SM = secondary metabolite, PSTF = pathway specific transcription factor, NRPS = nonribosomal peptide synthetase, HR-PKS = highly reducing polyketide synthase, NR-PKS = non-reducing polyketide synthase, TC = terpene cyclase. ^1^ No conserved domain means PSTF without conserved domain. ^2^ “x + x(+x)” means biosynthesis genes are not all located in a single cluster but in at least two chromosomal sites. ^3^ Two PSTFs are not located in a single cluster. ^4^ This domain is a fungal transcription factor regulatory middle homology region, which is present in the large family of fungal zinc cluster TFs. The regulatory function of this type of region is still unclear. ^5^ Gene designations are based on those from *Penicillium aethiopicum* [67].

**Table 2:** Known secondary metabolites and their pathway specific transcription factors in *A. fumigatus*.

SM	Backbone Gene	Backbone Gene Type	Gene Number	PSTF Number	PSTF	PSTF Type	Reference
DHN-melanin ^1^	*pksP*/*alb1* (Afu2g17600)	NR-PKS	6	0	/	/	[70,71]
Endocrocin/trypacidin	*encA* (Afu4g00210), *tpcC* (Afu4g14560)	NR-PKS, NR-PKS	4 + 13 ^2^	2	*tpcE, tpcD*	Zn(II)_2_Cys_6_, no conserved domain ^3^	[72]
Ferricrocin/TAFC ^4^	*sidC* (Afu1g17200), *sidD* (Afu3g03420)	NRPS, NRPS	1 + 1 + 3 ^2^	0	/	/	[73,74]
Fumagillin	*fmaB*/*fma-PKS* (Afu8g00370)	HR-PKS	10	1	*fapR*/*fumR*	Zn(II)_2_Cys_6_	[75,76,77]
Fumigaclavine C	*fgaPT1* (Afu2g17990), *pes1*/*pesB* (Afu1g10380), *pesL*/*fqzC* (Afu6g12050)	DMATS, NRPS, NRPS	11 + 1 + 1 ^2^	0	/	/	[32,33]
Fumigermin	*fgnA* (Afu1g01010)	PR-PKS	5	0	/	/	[28]
Fumihopaside A and B	*afumA* (AFUB_071550)	TC	4	1	*afumD*	Zn(II)_2_Cys_6_	[29]
Fumiquinazolines	*pesM* (Afu6g12080)	NRPS	4	0	/	/	[33,78]
Fumisoquins/fumipyrrole	*fsqF*/*fmpE* (Afu6g03480)	NRPS-like	7	1	*fsqA*/*fmpR*	Zn(II)_2_Cys_6_	[79,80]
Fumitremorgin/brevianamide F	*ftmA* (Afu8g00170)	NRPS	9	0	/	/	[81,82]
Gliotoxin	*gliP* (Afu6g09660)	NRPS	12 + 1 + 1 ^2^	1 + 1 ^5^	*gliZ*, *rglT*	Zn(II)_2_Cys_6_, Zn(II)_2_Cys_6_	[83,84,85,86]
Helvolic acid	*helA* (Afu4g14770)	TC	9	0	/	/	[87]
Hexadehydroastechrome	*hasD*/*pesF* (Afu3g12920), *hasE* (Afu3g12930)	NRPS, DMATS	8	2	*hasA, hasF*	Zn(II)_2_Cys_6_, Zn(II)_2_Cys_6_	[88]
Neosartoricin/fumicyclines	*nscA*/*fccA* (Afu7g00160)	NRPS	6	1	*nscR*	Zn(II)_2_Cys_6_	[89,90,91]
Psecurotin A	*posA* (Afu8g00540)	PKS/NRPS hybrid	6	1	*fapR*/*fumR*	Zn(II)_2_Cys_6_	[77,92]
Pyomelanin	*hppD* (Afu2g04200) ^6^	/	6	1	*hmgR*	Zn(II)_2_Cys_6_	[30,93]
Pyripyropene A	*pyr2* (Afu6g13930)	HR-PKS	8	0	/	/	[94]
Xanthocillin	*xanB* (Afu5g02660)	ICS	6	1	*xanC*	bZIP	[16]

SM = secondary metabolite, PSTF = pathway specific transcription factor, NRPS = nonribosomal peptide synthetase, HR-PKS = highly reducing polyketide synthase, NR-PKS = non-reducing polyketide synthase, PR-PKS = partially reducing polyketide synthase, DMATS = dimethylallyltryptophan synthase, TC = terpene cyclase, ICS = isocyanide synthase. ^1^ DHN-melanin = dihydroxynaphthalene melanin. ^2^ “x + x(+x)” means biosynthesis genes are not all located in a single cluster but in at least two sites. ^3^ No conserved domain means PSTF without conserved domain. ^4^ TAFC = triacetylfusarinine C. ^5^ Two PSTFs are not located in a single cluster. ^6^ Key enzyme gene involved in the L-tyrosine degradation pathway encoding 4-hydroxyphenylpyruvate dioxygenase.

**Table 3:** Occurrence of PSTF in different size of BGCs in *A. nidulans* and *A. fumigatus*.

	BGCs	PSTF-Containing BGCs
*Aspergillus nidulans*		
In total	28	12
Genes ≤ 5	13	0
Genes > 5	15	12
*Aspergillus fumigatus*		
In total	18	10
Genes ≤ 5	4	1
Genes > 5	14	9

BGCs = biosynthetic gene clusters, PSTF = pathway specific transcription factor.

**Table 4:** Different cases in *A. nidulans* and *A. fumigatus* of distribution of PSTF in BGCs.

Case Type	*Aspergillus nidulans*	*Aspergillus fumigatus*	Case in Total
One PSTF per BGC	6 (6 PSTFs + 6 BGCs) ^1^	5 (5 PSTFs + 5 BGCs) ^1^	11
Two PSTFs per BGC	4 (8 PSTFs + 4 BGCs) ^1^	2 (4 PSTFs + 2 BGCs) ^1^	6
One PSTF for two BGCs	/	1 (1 PSTF + 2 BGCs) ^1^	1
Additional case ^2^	1 (2 PSTFs + 2 BGCs) ^1^	1 (2 PSTFs + 1 BGC) ^1^	2

BGC = biosynthetic gene cluster, PSTF = pathway specific transcription factor. ^1^ Number of cases (number of PSTFs + number of BGCs involved). ^2^ Additional case indicates two more complex examples: (1) two PSTFs involved in a cross talk between two BGCs; (2) two PSTFs involved in one BGC with one PSTF located outside of the cluster.

## Data Availability

Not applicable.
